# Supplementary material for: Yap regulates skeletal muscle fatty acid oxidation and adiposity in metabolic disease
Source: Nat Commun. 2021 May 17;12:2887. doi: 10.1038/s41467-021-23240-7 (PMC8129430; doi:10.1038/s41467-021-23240-7)
Supplement: Supplementary file 1 — Supplementary Information [file 41467_2021_23240_MOESM1_ESM.pdf]

1 **Supplementary Information File**

a

Lane Number	Phenotype
1	Obese insulin resistant
2	Obese insulin resistant
3	Obese insulin resistant
4	Obese insulin resistant
5	Obese insulin resistant
6	Obese insulin sensitive
7	Obese insulin resistant
8	Obese insulin resistant
9	Obese insulin sensitive
10	Obese insulin sensitive
11	Obese insulin resistant
12	Obese insulin sensitive
13	Obese insulin resistant
14	Obese insulin resistant
15	Obese insulin resistant
16	Obese insulin resistant
17	Obese insulin resistant
18	Obese insulin resistant
20	Obese insulin resistant
21	Obese insulin resistant
22	Obese insulin resistant
23	Obese insulin sensitive
24	Obese insulin resistant
25	Obese insulin sensitive
26	Obese insulin sensitive
27	Obese insulin sensitive
28	Obese insulin sensitive
29	Obese insulin resistant
30	Obese insulin resistant
31	Obese insulin resistant
32	Obese insulin sensitive
33	Obese insulin resistant
34	Obese insulin resistant
35	Obese insulin resistant
38	Obese insulin sensitive
39	Obese insulin resistant
40	Obese insulin sensitive
41	Obese insulin sensitive
42	Obese insulin resistant
43	Obese insulin resistant
44	Obese insulin resistant
45	Obese insulin resistant
46	Obese insulin sensitive
47	Obese insulin resistant
48	Obese insulin resistant
49	Obese insulin resistant
50	Obese insulin sensitive
51	Obese insulin resistant
52	Obese insulin resistant
53	Obese insulin sensitive
54	Obese insulin sensitive
55	Obese insulin sensitive
56	Obese insulin resistant
57	Obese insulin resistant

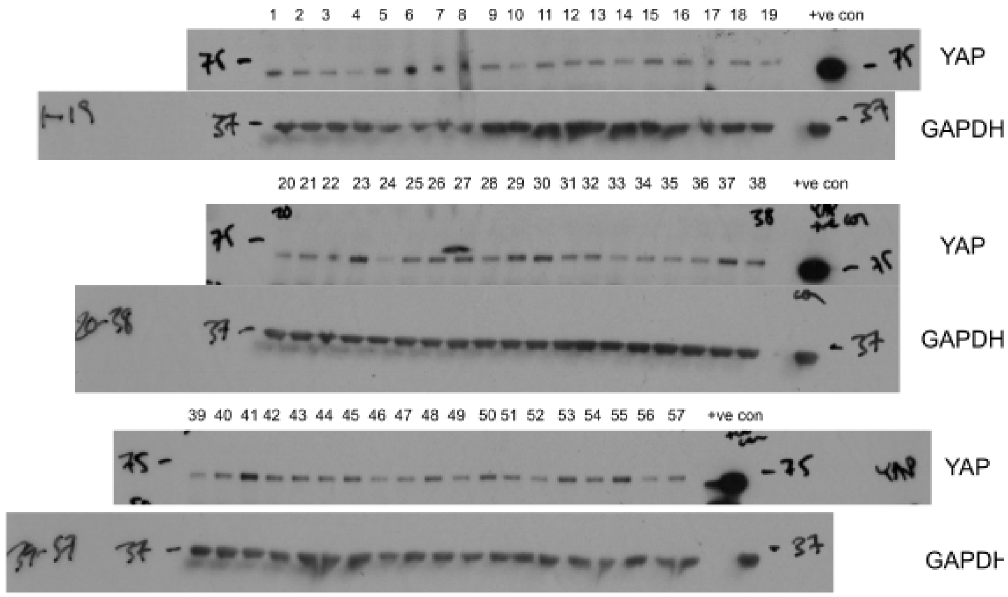

b

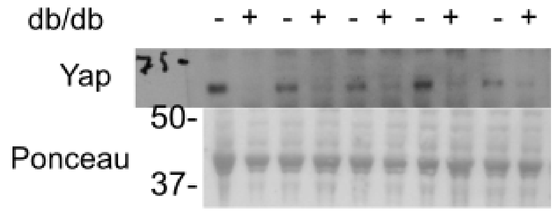

c

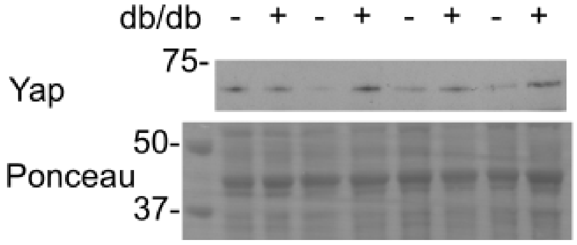

3 **Supplementary Fig 1.** Uncropped Western blots for data shown in Fig 1. Uncropped blots from data shown in A) Fig 1B-D, B) Fig 1F-H.

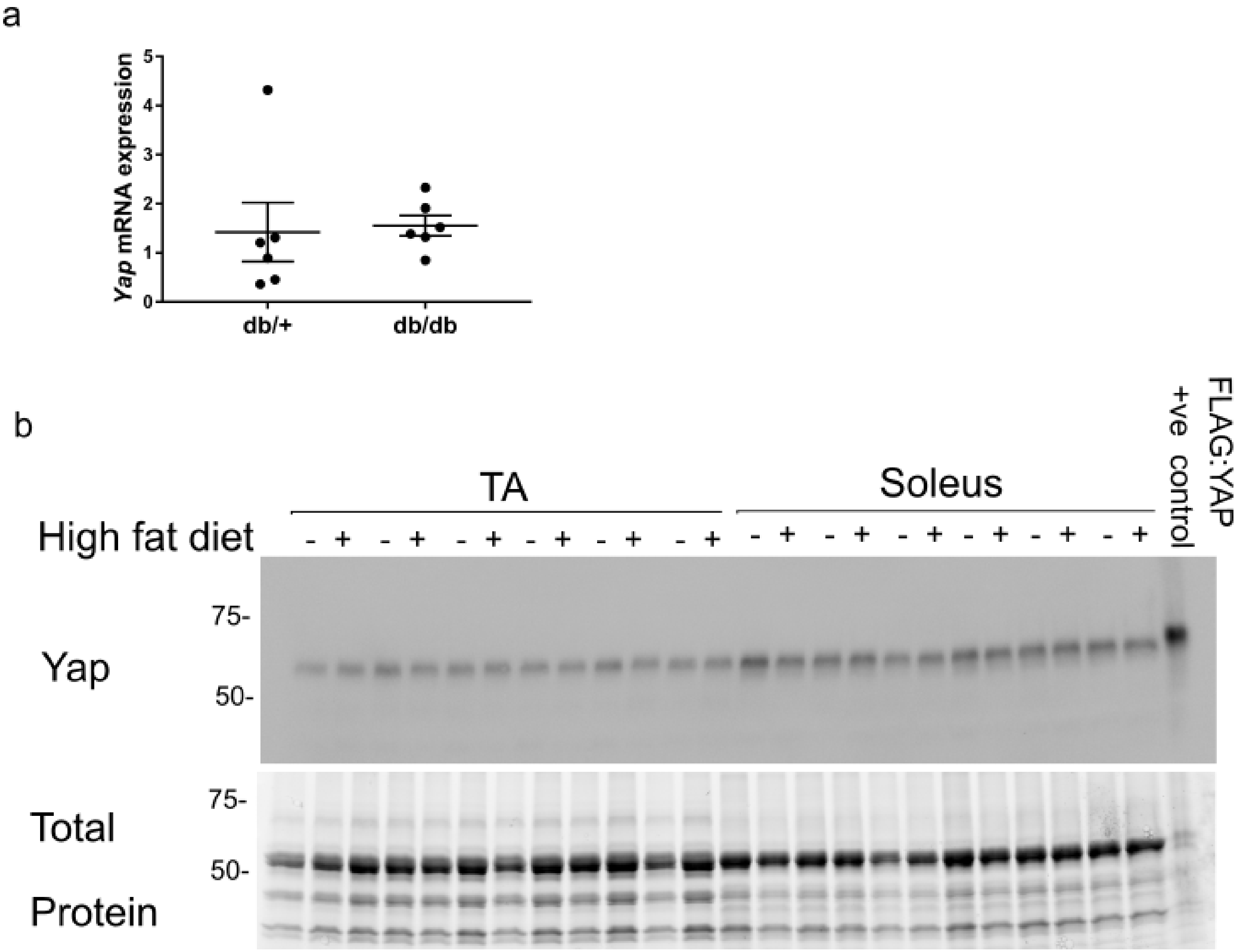

Supplementary Fig 2. *Yap* mRNA expression in *db/db* tibialis anterior (TA) and soleus muscles from a diet-induced mouse model of obesity. A) *Yap* mRNA expression in *db/db* and *db/+* TA muscle (n=6 biologically independent animals, mean ± SEM, p=0.84, two sided t-test), B) Western blots of total Yap and total protein levels in TA and soleu*s* muscles of C57BL/6J mice fed standard chow or 43 % high-fat diet for 6 weeks (n=6 biologically independent animals).

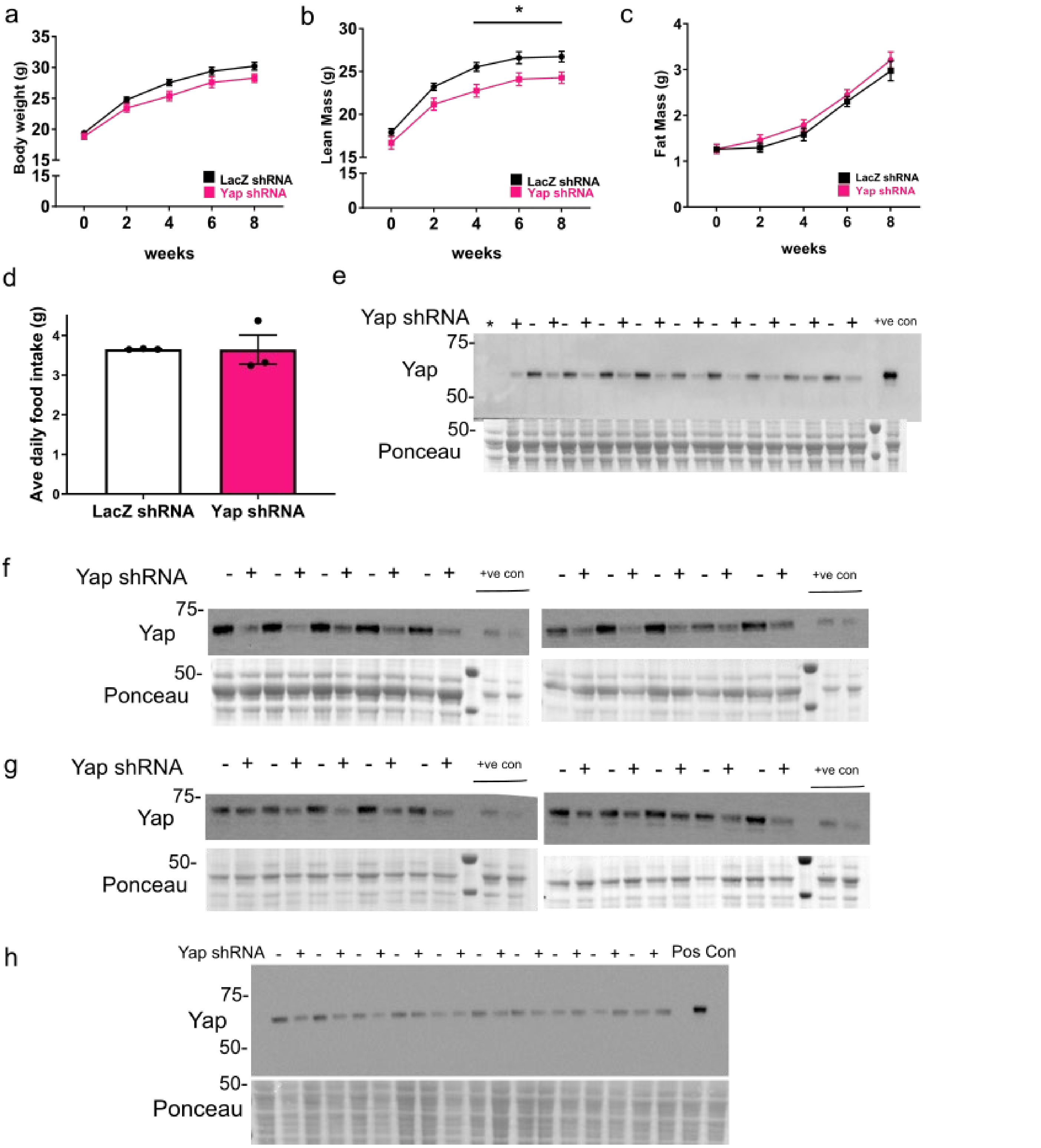

Supplementary Fig 3. Yap knockdown causes a reduction in mass in limb skeletal muscles with different composition of fiber types and intrinsic metabolic properties. A) Body mass, B) lean mass and C) fat mass determined by quantitative magnetic resonance for C57BL/6J mice administered an intravenous injection of AAV6:lacZ shRNA (black) or AAV:Yap-shRNA (pink) vectors at $1x10^{12}$ vg at 5 weeks of age (n=10 biologically independent animals, mean ± SEM, * indicates = p<0.05, two way ANOVA with Sidaks multiple comparison test, exact p-values presented in source data), D) Average daily food intake at 6 weeks post treatment in animals treated as in Supplementary Fig 3A-C (n=3 daily measurements taken from 10 biologically independent animals per treatment group, mean ± SEM, two sided t-test, p=0.98), E-H) Western blot of total Yap protein in E) TA (* indicates sample not quantified due to variation in total protein content), F) Gastroc, G) soleus muscles and H) liver of mice treated as in A) at experimental endpoint (n=10 biologically independent animals for all muscles except TA (E) where n=9 lacZ shRNA treated and 10 Yap shRNA treated).

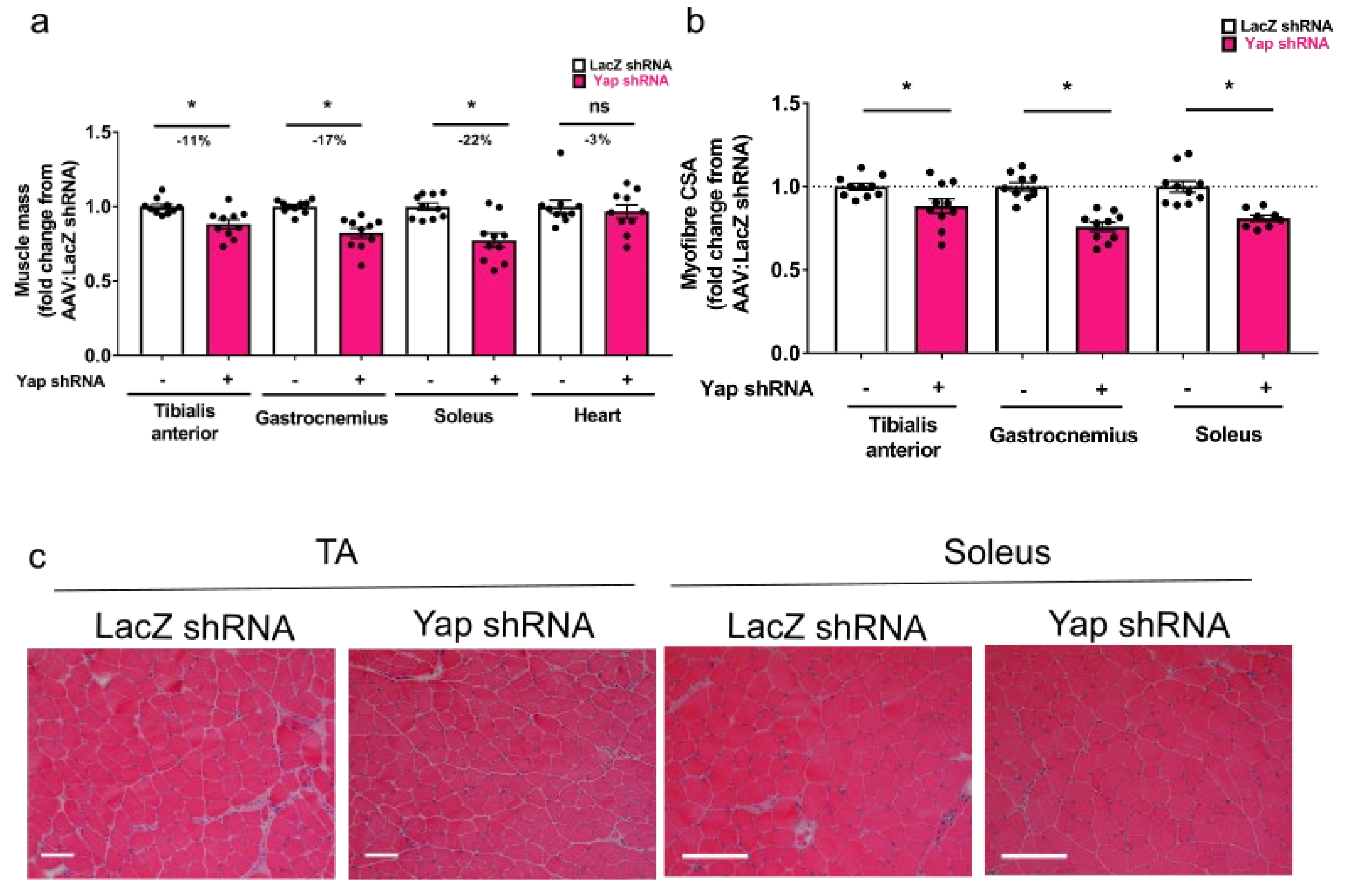

Supplementary Fig 4. Yap regulates adult skeletal muscle mass regardless of metabolic phenotype. A) Muscle mass in TA, Gastroc, Soleus and Heart of mice treated as in Supplementary Fig 3) (n=10 biologically independent animals, mean ± SEM, * indicates sig difference between conditions, p=0.003, 0.0001, 0.0005 and 0.6 respectively, two-sided t-test), B) Cross-sectional area (CSA) of muscle fibers from muscles treated as in Supplementary Fig 3 (n=10, except soleus Yap shRNA where n=9 biologically independent animals, mean ± SEM, two sided t-test, * indicates sig difference between conditions, p=0.02, <0.0001, 0.0002 respectively), C) Representative sections of TA and soleus muscle morphology stained with Haematoxylin and Eosin. (n=10 biologically independent animals, scale bar indicates 50 μm).

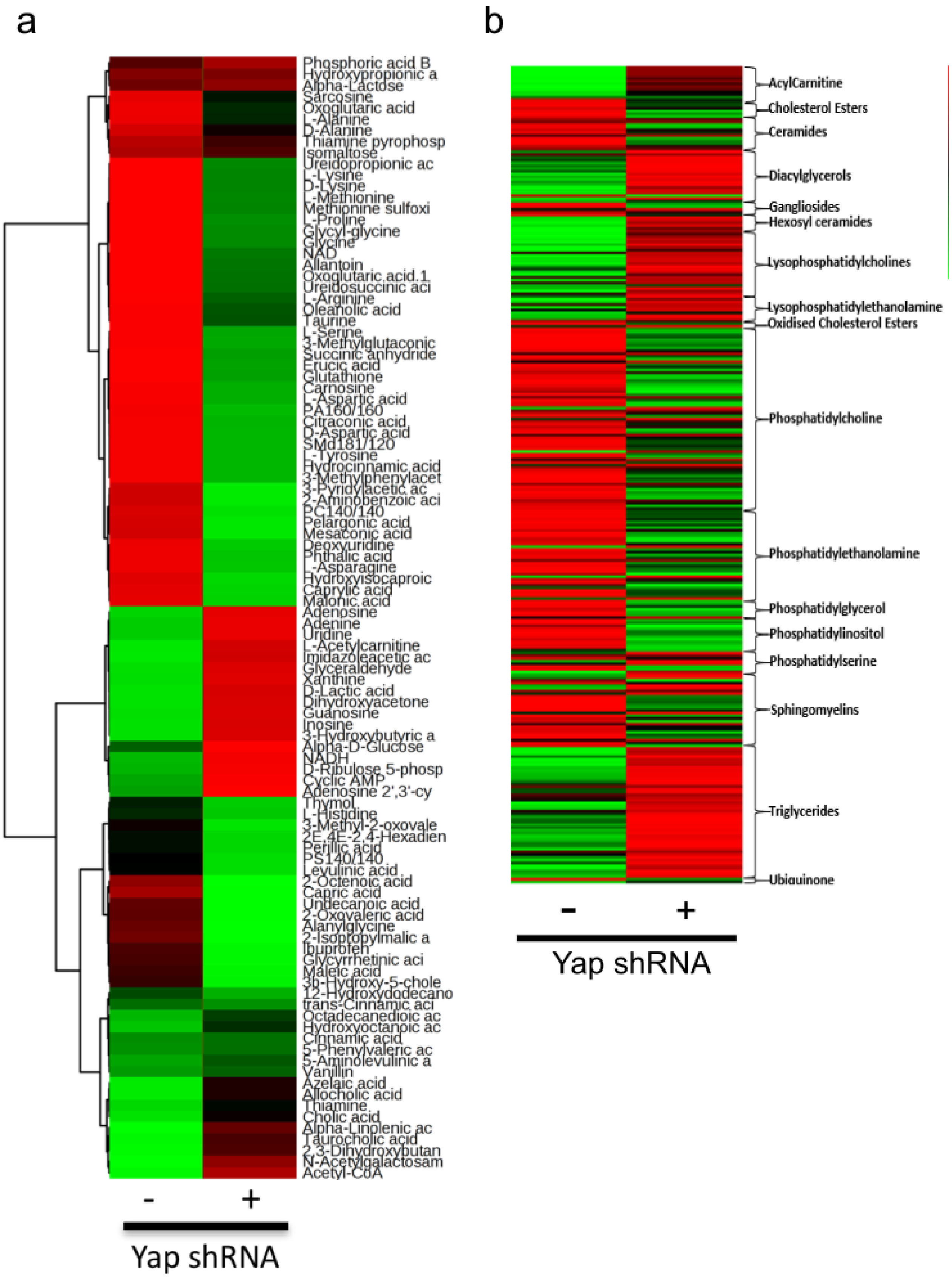

Supplementary Fig 5. Yap regulates adult skeletal muscle mass regardless of metabolic phenotype. A) Hierarchical-clustered heat map showing the top 100 metabolite features ranked by t-test value (red=lower p value and green = higher p value) of mouse soleus muscles examined 4 weeks after injection of AAV6:lacZ shRNA or AAV6:Yap shRNA vectors (n=5 lacZ shRNA (-) and 6 Yap shRNA (+) biologically independent animals), B) Hierarchical-clustered heat map showing the differential expression of lipid classes ranked by t-test (red=lower p value and green = higher p value, n=6 biologically independent animals).

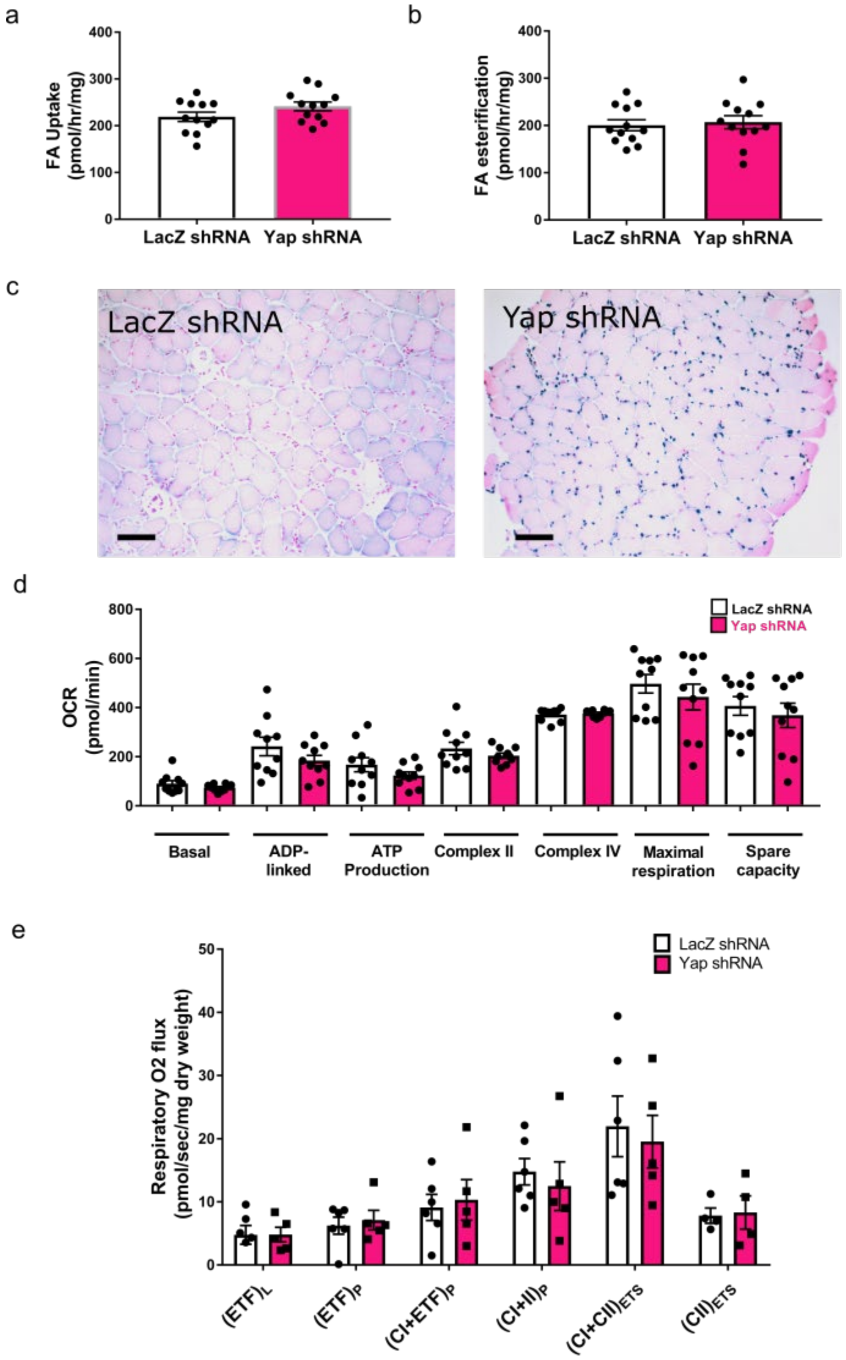

Supplementary Fig 6. Yap knockdown does not alter fatty acid uptake or esterification or directly impact mitochondrial respiratory capacity but leads to marked DNA fragmentation of myonuclei. A) ^{14}C Oleate uptake (pmol/hr/mg tissue) in AAV6:LacZ shRNA and AAV6:Yap shRNA vector treated EDL muscles at 2 weeks post injection (n=12 biologically independent animals, mean ± SEM, two-sided t-test, p=0.12), B) Fatty acid esterification rate (pmol/hr/mg) in AAV6:lacZ shRNA and AAV6:Yap shRNA vector treated EDL muscles at 2 weeks post injection (n=12 biologically independent animals, mean ± SEM, two-sided t-test, p=0.71), C) Representative images of TUNEL positive (black) nuclei in soleus muscles treated with AAV6:lacZ-shRNA and AAV6:Yap-shRNA and assessed 8 weeks after treatment (n=10 biologically independent animals, scale bars indicate 100 µm), D) Oxygen consumption rate (OCR, pmol/min) during Seahorse respiratory stress test in isolated mitochondria (5 ng/well) from gastrocnemius of mice treated by intravenous injection with AAV6:lacZ shRNA (white) or AAV6:Yap shRNA vectors (pink) at $1x10^{12}$ vg from 5 weeks of age for 8 weeks (n=10 biologically independent animals, mean ± SEM, two-sided t-test, exact p values in source data), E) Assessment of respiratory capacity (pmol/sec/mg dry tissue) using Ouroboros respirometer in intact EDL limb muscles at 4 weeks following treatment with AAV-lacZ shRNA (white) or AAV-Yap shRNA (pink) (n=6 LacZ shRNA and 5 YAP shRNA biologically independent animals except for $(CII)_{ETS}$ where n=4, mean ± SEM, two sided t-test, exact p values in source data).

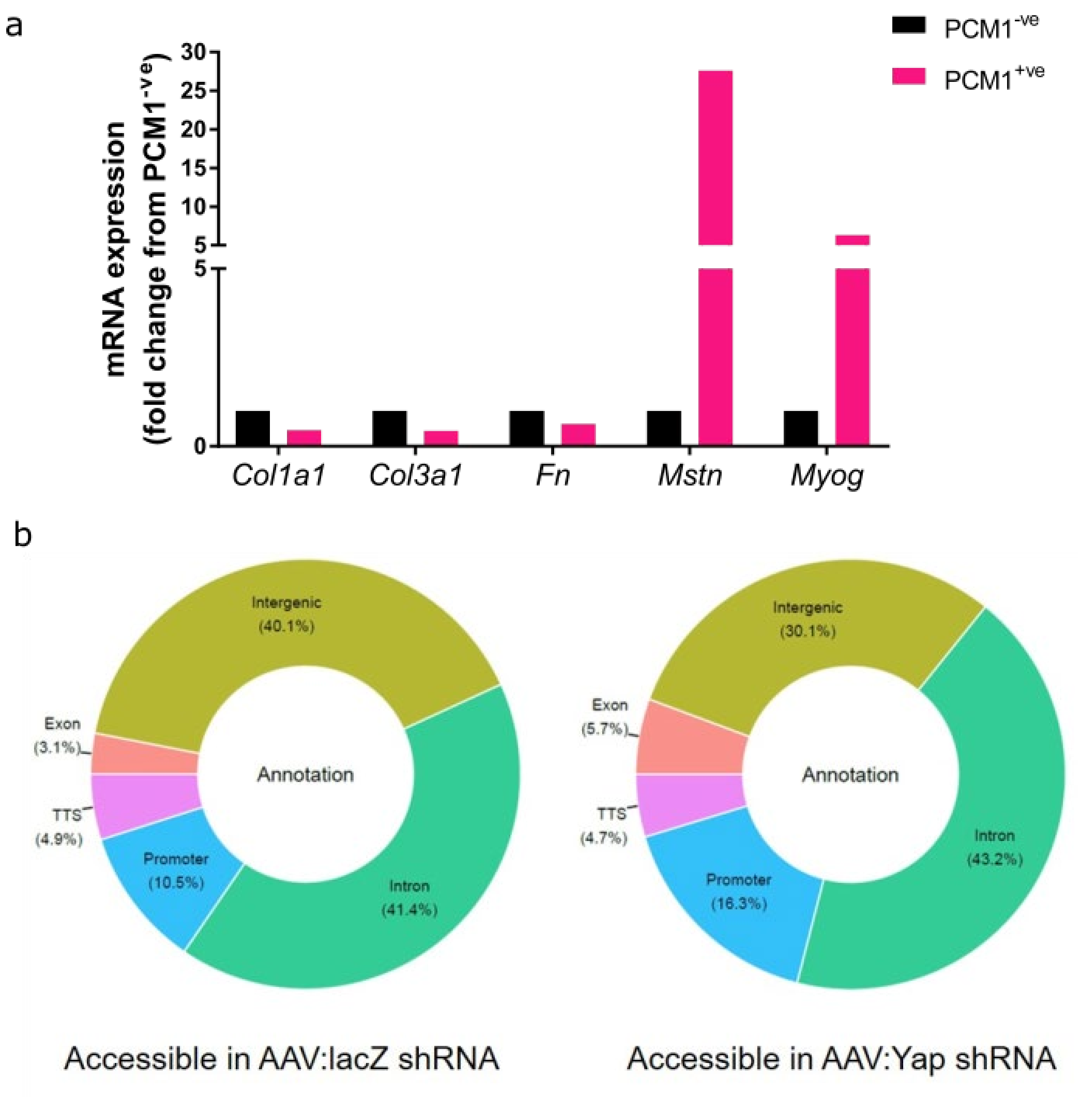

Supplementary Fig 7. Validation of PCM1 myonuclei sorting and distribution of changes in chromatin accessibility. A) q-PCR expression of fibroblast markers (*Col1a1, Col3a1, Fn*) are lower, while myogenic cell markers (*Myog* and *Mstn*) are more abundant in PCM1 sorted pooled nuclei from adult limb muscles highlighting successful isolation of the myonuclei population (n=1 from nuclei pooled from 3 treated Gastroc muscles from biologically independent animals), B) Piecharts showing the genomic distribution of differentially expressed peaks following ATAC-sequencing in AAV6-lacZ shRNA and AAV6-Yap shRNA treated limb muscles at 28 days post treatment.

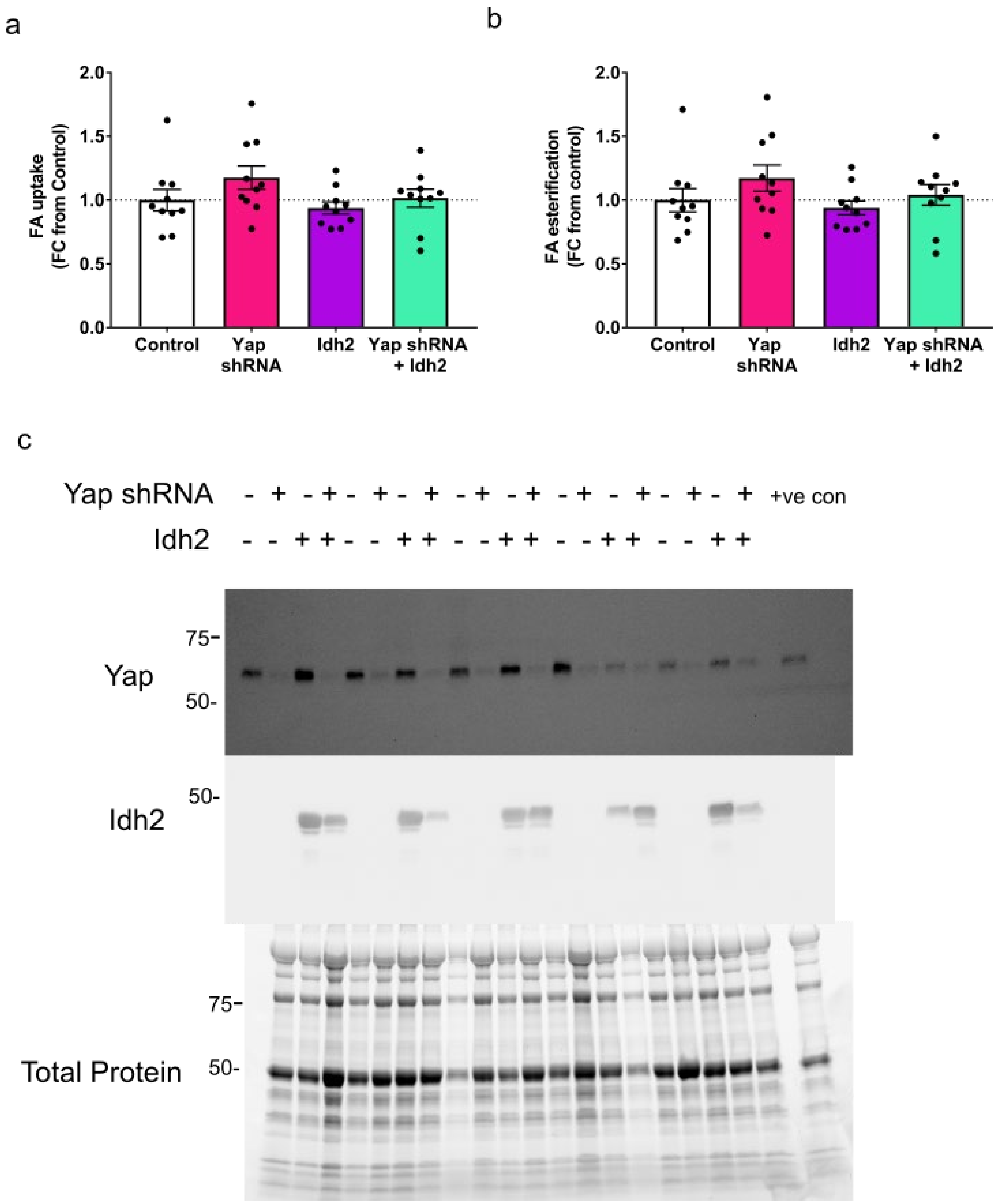

Supplementary Fig 8. Yap knockdown and Idh2 expression does not affect fatty acid uptake or esterification. A) A) ^{14}C Oleate uptake (pmol/hr/mg tissue) in control, AAV6:Yap shRNA, AAV6:Idh2 vector or both treated EDL muscles at 2 weeks post injection (n=10 biologically independent animals, mean ± SEM, one-way ANOVA with Tukey multiple comparison test, exact p values shown in source data), B) Fatty acid esterification rate (pmol/hr/mg) in muscles treated as in A) (n=10 biologically independent animals, mean ± SEM, one-way ANOVA with Tukey's multiple comparison test, exact p values in source data), C) Western blots of Yap, Idh2 and total protein from TA muscles of mice treated as in A) (n=5 biologically independent animals).

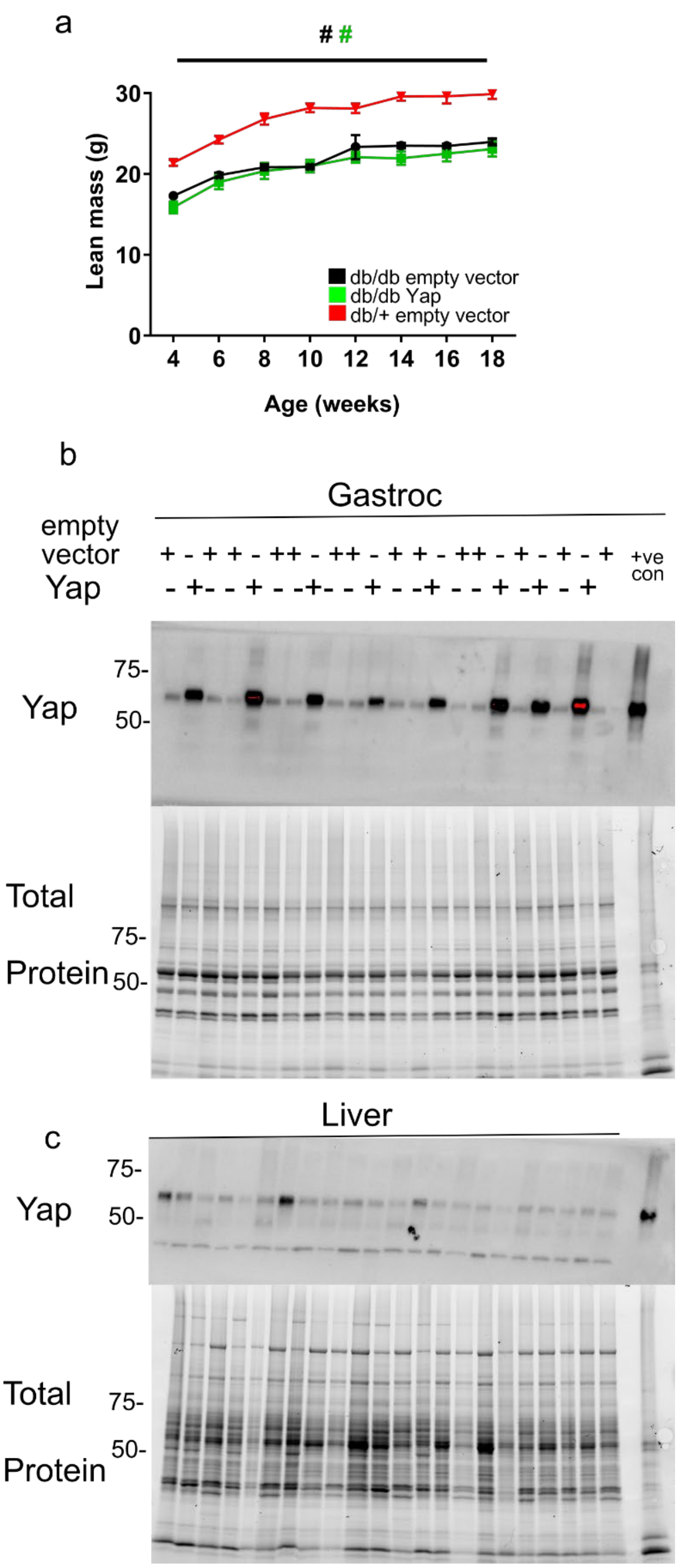

Supplementary Fig 9. Striated muscle expression of Yap limits adiposity without altering lean mass in *db/db* mice. A) Lean mass (in grams) as determined by quantitative magnetic resonance analysis in *db/db* mice administered AAV6:empty vector (black), db/db mice treated with AAV6:Yap vector (green) or *db/+* mice administered AAV6:empty vector (red) between 4 and 18 weeks of age (n=7 *db/db* empty vector, 9 *db/db* Yap, 8 *db/+* empty vector biologically independent animals, mean ± SEM, * indicates sig difference between conditions, two-way ANOVA with Tukey’ multiple comparison test, exact p values reported in source data), B) Western blots of Yap levels in gastrocnemius and C) liver of *db/+* (-) or *db/db* (+) mice examined 14 weeks after treated by intravenous injection with AAV6:empty vector (-) or AAV6:Yap (+) vector (n=6 *db/db* empty vector, 8 *db/db* Yap, 8 *db/+* empty vector biologically independent animals).

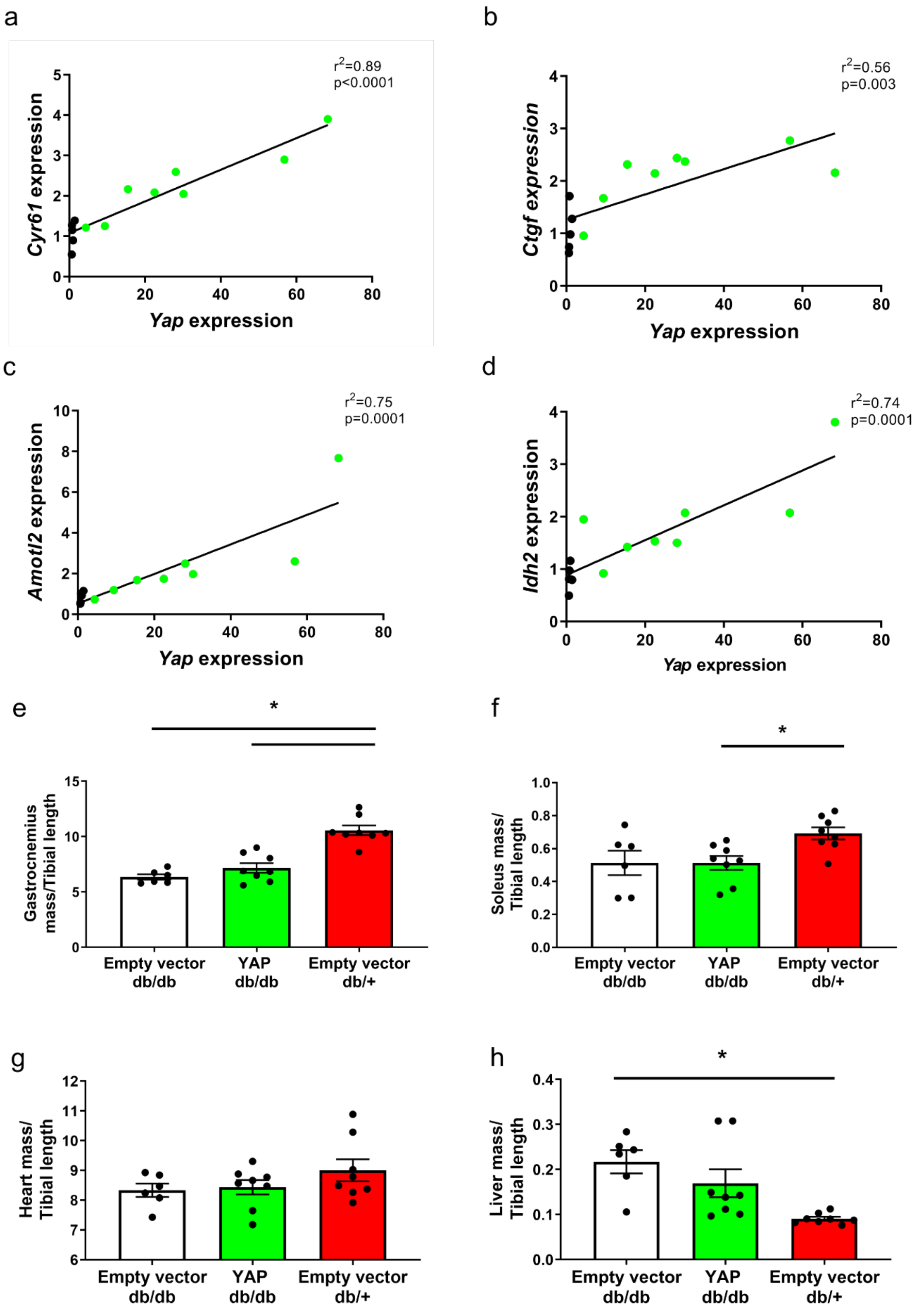

Supplementary Fig. 10 Yap target gene expression in *db/db mice* and endpoint tissue weights. qPCR assessment of *Yap* mRNA expression relative to A) *Cyr61,* B) *Ctgf,* C) *Amotl2* and D*) Idh2* mRNA expression in Gastroc muscles of *db/db* mice treated with AAV-empty vector or AAV-Yap at experimental endpoint (n=5 *db/db* empty vector (black) and 8 *db/db* Yap (green) biologically independent animals, mean ± SEM, Pearson's correlation test, p values shown on graphs and in source data), E) Gastrocnemius, F) Soleus, G) Heart and H) Liver mass normalised to tibial length in mice treated as in A) (n=6 *db/db* empty vector (white), 8 *db/db* Yap (green), 8 *db/+* empty vector (red) biologically independent animals, mean ± SEM, * indicates sig difference between conditions, one-way ANOVA with Tukey' multiple comparison test, exact p values reported in source data).

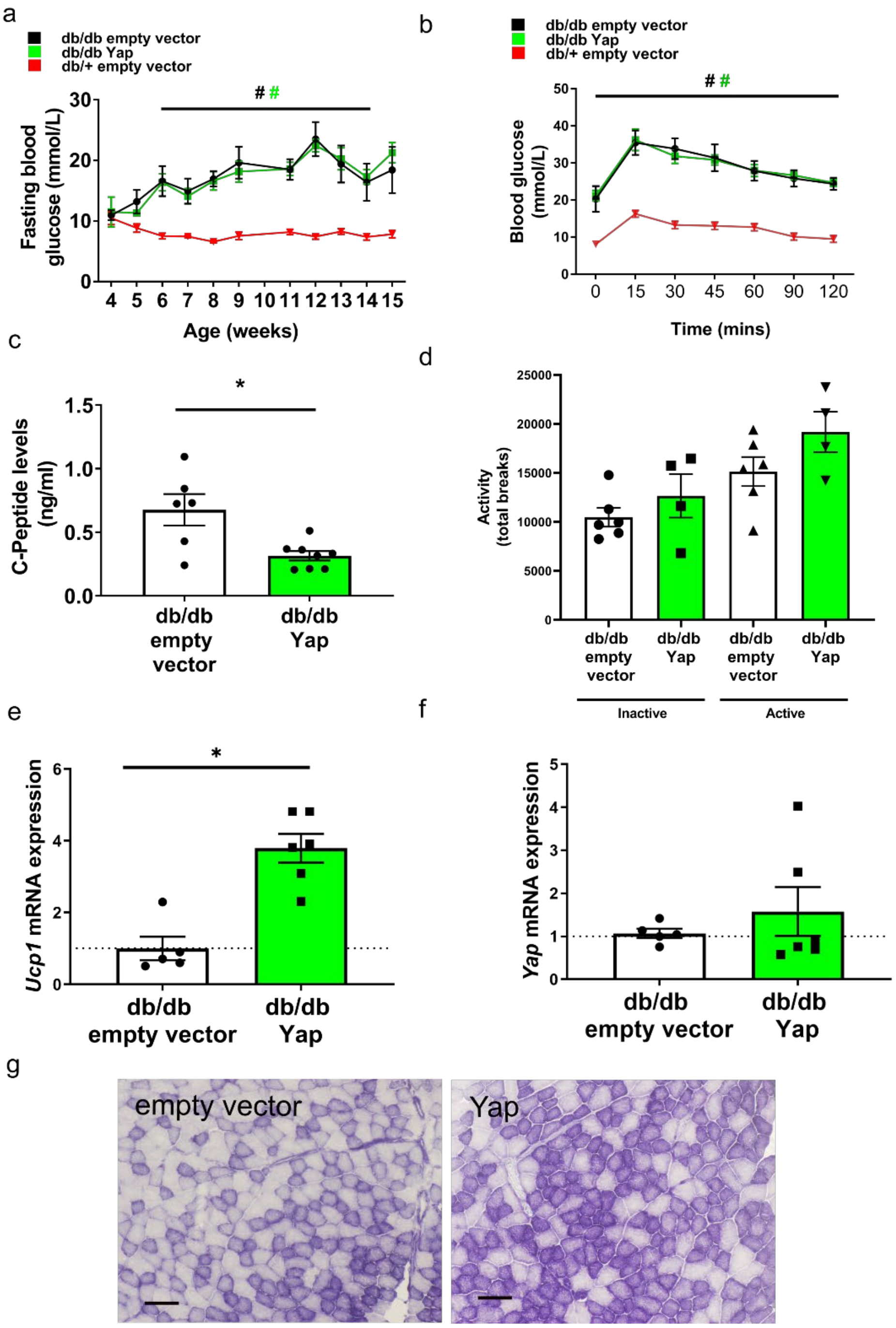

Supplementary Fig 11. Characterisation of metabolic feature of *db/db* mice expressing AAV6:Yap in striated muscle. A) Fasting blood glucose from 4-15 weeks of age in mice treated as in Supplementary Figure 9, (n=7 *db/db* empty ector (black), 9 *db/db* Yap (green), 8 *db/+* empty vector (red) biologically independent animals, mean ± SEM, * indicates sig difference between conditions, two-way ANOVA with Tukey' multiple comparison test, exact p values reported in source data), B) Oral Glucose tolerance test of mice treated as in Supplementary Figure 9, (n=7 *db/db* empty vector (black), 9 *db/db* Yap (green), 8 *db/+* empty vector (red) biologically independent animals, mean ± SEM, * indicates sig difference between conditions, two-way ANOVA with Sidak's multiple comparison test, exact p values reported in source data), C) C-peptide levels (ng/ml) in mice treated as in B) (n=6 *db/db* empty vector, 8 *db/db* Yap biologically independent animals, mean ± SEM, two-sided t-test, * indicates sig difference between conditions, p=0.008), D) total activity levels of mice treated as in Supplementary Fig 9 after 10 weeks of treatment (n=6 *db/db* empty vector and 4 *db/db* Yap biologically independent animals, mean ± SEM, two-sided t-test, p=0.33 inactive phase and 0.14 active phase), E) *Ucp1* and F) *Yap* mRNA expression in inguinal adipose tissues of mice treated as in Supplementary Figure 9 (n=5 *db/db* empty vector and 6 *db/db* Yap, mean ± SEM, two-sided t-test, p=0.0005 and p=0.44 respectively), G) Representative image of TA muscle labelled for succinate dehydrogenase activity (SDH) activity (n=6 biologically independent animals, scale bar indicates 50 µm).
