# Supplementary material for: Yap regulates skeletal muscle fatty acid oxidation and adiposity in metabolic disease
Source: Nat Commun. 2021 May 17;12:2887. doi: 10.1038/s41467-021-23240-7 (PMC8129430; doi:10.1038/s41467-021-23240-7)
Supplement: Supplementary file 3 — Description of Additional Supplementary Files [file 41467_2021_23240_MOESM3_ESM.pdf]

Description of Additional Supplementary Files

File Name: Supplementary Data 1

Description: Metabolomics and Lipidomics datasets from data shown in Fig 2.

File Name: Supplementary Data 2

Description: ATAC-sequencing dataset from data shown in Fig 3.

File Name: Supplementary Data 3

Description: RNA-sequencing dataset from data shown in Fig 3.

File Name: Supplementary Data 4

Description: GSEA analysis of RNA-sequencing dataset shown in Fig 3.

File Name: Supplementary Data 5

Description: Proteomics dataset from data shown in Fig 3.

File Name: Supplementary Data 6

Description: GSEA analysis of Proteomics data shown in Fig 3.

File Name: Supplementary Data 7

Description: Primer and Probe IDs for Taqman assays or Primer sequences for Syber Green assays used in this study.
