# Supplementary material for: Yap regulates skeletal muscle fatty acid oxidation and adiposity in metabolic disease
Source: Nat Commun. 2021 May 17;12:2887. doi: 10.1038/s41467-021-23240-7 (PMC8129430; doi:10.1038/s41467-021-23240-7)
Supplement: Supplementary file 11 — Reporting Summary [file 41467_2021_23240_MOESM11_ESM.pdf]

nature research

Corresponding author(s): A/Prof. Paul Gregorevic

Last updated by author(s): Mar 1, 2021

Reporting Summary

Nature Research wishes to improve the reproducibility of the work that we publish. This form provides structure for consistency and transparency in reporting. For further information on Nature Research policies, see our Editorial Policies and the Editorial Policy Checklist.

Statistics

For all statistical analyses, confirm that the following items are present in the figure legend, table legend, main text, or Methods section.

n/a	Confirmed	
☐	☒	The exact sample size (*n*) for each experimental group/condition, given as a discrete number and unit of measurement
☐	☒	A statement on whether measurements were taken from distinct samples or whether the same sample was measured repeatedly
☐	☒	The statistical test(s) used AND whether they are one- or two-sided<br>*Only common tests should be described solely by name; describe more complex techniques in the Methods section.*
☐	☒	A description of all covariates tested
☐	☒	A description of any assumptions or corrections, such as tests of normality and adjustment for multiple comparisons
☐	☒	A full description of the statistical parameters including central tendency (e.g. means) or other basic estimates (e.g. regression coefficient) AND variation (e.g. standard deviation) or associated estimates of uncertainty (e.g. confidence intervals)
☐	☒	For null hypothesis testing, the test statistic (e.g. *F*, *t*, *r*) with confidence intervals, effect sizes, degrees of freedom and *P* value noted<br>*Give P values as exact values whenever suitable.*
☒	☐	For Bayesian analysis, information on the choice of priors and Markov chain Monte Carlo settings
☒	☐	For hierarchical and complex designs, identification of the appropriate level for tests and full reporting of outcomes
☐	☒	Estimates of effect sizes (e.g. Cohen's *d*, Pearson's *r*), indicating how they were calculated

Our web collection on statistics for biologists contains articles on many of the points above.

Software and code

Policy information about availability of computer code

Data collection

No software was used for data collection.

Data analysis

Data in the manuscript were analyzed using Prism v8 and v9 with the following exceptions:

ATM.-sequencing and RNA-sequencing experiments were analyzed using Skewer (Skewar 0.2.2).Reads were mapped with STAR aligner (STAR 2.5.3a) and BWA (BWA0.7.17) for RNA and ATAC assays respectively. Duplicated reads were removed from ATAC-seq datasets with Samtools (v1.8) and peak calling was performed with MACS 1.4.2-1. A peak set was curated with Featurecounts (v1.5.0). Data was compared with EdgeR (v3.16.5 and v3.26.8). GSEA (4.1.0) was used for gene set enrichment analysis. Enhancer/Promoter analysis was performed with MITCH (v3.12). TEAD binding sites in ATAC peaks at enhancers were identified with Bedtools (v2.28.0) based shell script and Bedtools genomecov tool was used to convert BAM files to bedgraph format so that sequencing coverage plots could be generated in R (v4.0.2) with rtracklayer (v1.50.0) and a custom R script. All code used in the analysis of data and generation of related figures is accessible via https://github.com/markziemann/watt2021_yap and https://evangelynsim.github.io/2021_UoM_Yap_shRNA_nuclei_RNAseq_ATACseq/

Proteomics mass spec data was processed with Andromeda/MaxQuant using the UniProt database (June 2019) and Max LFQ algorithm (v1.6.2.2).

Quantification of Western blots was performed using ImageJ (1.53c).

Intact muscle respiration was analyzed using Datalab2 software (Oroboruos Instruments v7.4).

Respiration in isolated mitochondria was analyzed using Wave (v2.6).

Metabolite peak calling and quality check was performed using QTOF Masshunter Quant software (Agilent v5.1). Heatmaps were generated using MetaboAnalyst (v4.0).

For manuscripts utilizing custom algorithms or software that are central to the research but not yet described in published literature, software must be made available to editors and reviewers. We strongly encourage code deposition in a community repository (e.g. GitHub). See the Nature Research guidelines for submitting code & software for further information.

Data

Policy information about availability of data

All manuscripts must include a data availability statement. This statement should provide the following information, where applicable:

- Accession codes, unique identifiers, or web links for publicly available datasets
- A list of figures that have associated raw data
- A description of any restrictions on data availability

ATAC-seq and RNA-seq data reported in this manuscript have been deposited in the NCBI Gene Expression Omnibus and are accessible through the GEO Series accession number GSE144138 (https://www.ncbi.nlm.nih.gov/geo/query/acc.cgi?acc=GSE144138). The mass spectrometry proteomics data have been deposited to the ProteomeXchange Consortium via the PRIDE partner repository with the dataset identifier PXD024379 (http://proteomecentral.proteomexchange.org/cgi/GetDataset?ID=PXD024379).

Field-specific reporting

Please select the one below that is the best fit for your research. If you are not sure, read the appropriate sections before making your selection.

[x] Life sciences [ ] Behavioural & social sciences [ ] Ecological, evolutionary & environmental sciences

For a reference copy of the document with all sections, see nature.com/documents/nr-reporting-summary-flat.pdf

Life sciences study design

All studies must disclose on these points even when the disclosure is negative.

Sample size	Samples sizes were determined using predicted effect size from previous work with an alpha value of 0.05 and a beta value of 0.1. Samples were biologically independent replicates with n values noted in the appropriate legends.
Data exclusions	No data exclusion criteria was used in this study.
Replication	Data was replicated successfully for all studies on at least 2 occasions with the following exceptions. The human study in Fig 1 was not independently replicated due to cost and ethical considerations although Western blots were were replicated on more than one occasion successfully.
Randomization	Animals were randomly assigned to experimental groups. For experiments with human subjects, randomisation was not possible due to subject characteristics and study design. Full details are provided in reference 73 and in the section "Human Research particpants" below.
Blinding	Investigators were blinded to allocation during experiments and to experimental outcome on at least one replication study.

Reporting for specific materials, systems and methods

We require information from authors about some types of materials, experimental systems and methods used in many studies. Here, indicate whether each material, system or method listed is relevant to your study. If you are not sure if a list item applies to your research, read the appropriate section before selecting a response.

Materials & experimental systems

n/a	Involved in the study	
[ ]	[x]	Antibodies
[ ]	[x]	Eukaryotic cell lines
[x]	[ ]	Palaeontology and archaeology
[ ]	[x]	Animals and other organisms
[ ]	[x]	Human research participants
[x]	[ ]	Clinical data
[x]	[ ]	Dual use research of concern

Methods

n/a	Involved in the study	
[x]	[ ]	ChIP-seq
[x]	[ ]	Flow cytometry
[x]	[ ]	MRI-based neuroimaging

Antibodies

Antibodies used	UCP-1 (1/1000; ab10983, Abcam, Cambridge, UK).<br>YAP (#14074), IDH2 (#56439) (1/1000, Cell Signalling Technologies)<br>GAPDH (#SC-32233) (1/20,000 SantaCruz Biotechnology).<br>Goat anti-rabbit secondary antibody (#1706516, Bio-Rad technologies, 1/5000 dilution).<br>Goat anti-mouse secondary antibody (#1706515, Bio-Rad technologies, 1/5000 dilution).
Validation	UCP1 antibody was used and validated previously (Dodd GT et al 2015 Cell). YAP, GAPDH and secondary antibodies were validated previously (Watt et al 2015 Nature Comms) and in this study using positive control samples (YAP overexpression and shRNA constructs). Idh2 antibody was validated in Fig 4 of this study using mouse limb muscles treated with AAV6 vectors encoding murine Idh2.

Eukaryotic cell lines

Policy information about cell lines

Cell line source(s)	Cells were originally sourced from American Type Culture Collection (ATCC).
Authentication	Cells were authenticated by visualization of cellular morphology, assessment of growth curves and assessment of mycoplasma contamination.
Mycoplasma contamination	Cells tested negative for mycoplasma contamination.
Commonly misidentified lines<br>(See ICLAC register)	N/A

Animals and other organisms

Policy information about studies involving animals; ARRIVE guidelines recommended for reporting animal research

Laboratory animals	Male C57BL/6 mice aged 4-10 weeks of age and B6.BKS(D)-Leprdb/J (db/db and db/+) male mice aged 4 -5 weeks were used in these studies. All mice were maintained in a 12:12 hr light:dark cycle at a temperature of 22 ± 1°C, a humidity of 40-70 %, and provided ad libitum access to food and water during the study duration.
Wild animals	No wild animals were used in these studies.
Field-collected samples	No field collected samples were used.
Ethics oversight	Animal experiments were conducted in accordance with the relevant codes of practice for the care and use of animals for scientific purposes as stipulated by the National Health and Medical Research Council of Australia and conducted with approval from the respective Animal Ethics Committees of the Alfred Medical Research and Education Precinct (AMREP, E/1659/2015/B), and the University of Melbourne (Melbourne, Australia, 1714353 and 1814680).

Note that full information on the approval of the study protocol must also be provided in the manuscript.

Human research participants

Policy information about studies involving human research participants

Population characteristics	64 subjects underwent a hyperinsulinemic-euglycemic clamp study (35 female (23 Insulin resistant group (IR)) and 29 male (20 IR)). Average age was 50 +/- 11 (IR) and 50 +/- 12.6 (Insulin Sensitive group; IS). Ave BMI was 37.1 +/- 4.8 (IR) and 35.3 +/- 4.2 (IS). 14 subjects had prediabetes. Of these, we obtained 57 independent samples for biochemical analysis.<br><br>Full subject characteristics are provided in Table 1 of the original study (Reference 73. Chen D.L. et al J Clin Endocrinol Metab (2015)).
Recruitment	Subjects (n=184) were recruited by advertisements in newspapers over an 18-month period (2011-2013). Written consent was obtained prior to study commencement. Screening assessed eligibility, including age (18-70 years) and body mass index (>30kg/m2). Exclusion criteria were diabetes; treatment with medications that impact glucose metabolism; alcohol intake >20g/d or 40g/d in females or males respectively; weight change >5 % in the 3 months prior to study commencement; known renal, cardiac or liver disease; and current cancers (number excluded = 104). Eligible subjects (n=80) underwent a 75-g oral glucose tolerance test (oGTT) using American Diabetes Association criteria to exclude subjects with undiagnosed diabetes (n=2). Fourteen subjects did not proceed to the full study due to loss of interest (n=4), illness (n=4), and difficult venous access (n=6). There were no differences in age, BMI, BP, waist circumference between these subjects and the final cohort. Subjects were instructed not to perform vigorous exercise, to abstain from alcohol, and to record their diet in the 2 days before the clamp. Subjects attended the research facility following an overnight fast. Premenopausal females had urine beta-human chorionic gonadotrophin levels checked to preclude pregnancy.
Ethics oversight	Human muscle biopsies were procured previously with ethical consent provided by the St Vincent's Hospital Human Research Ethics Committee (HREC/10/SVH/133. Sydney, Australia). All relevant ethical regulations were complied with and informed consent was provided by study participants.

Note that full information on the approval of the study protocol must also be provided in the manuscript.
